# Scaling Up the Family Integrated Care Model in a Level IIIC Neonatal Intensive Care Unit: A Systematic Approach to the Methods and Effort Taken for Implementation

**DOI:** 10.3389/fped.2021.682097

**Published:** 2021-06-09

**Authors:** Bárbara Moreno-Sanz, María Teresa Montes, Marta Antón, María Teresa Serrada, Marta Cabrera, Adelina Pellicer

**Affiliations:** ^1^Department of Neonatology, La Paz University Hospital, Madrid, Spain; ^2^Hospital La Paz Institute for Health Research-IdIPAZ, La Paz University Hospital, Madrid, Spain

**Keywords:** FICare, family integrated care, parent education, parent training, parent empowerment

## Abstract

**Background:** Family Integrated Care (FICare) integrates parents in the direct care of their child while the healthcare personnel act as teachers and guides. To this date, most reports on the feasibility of this model refer to stable preterm infants admitted to Neonatal Intensive Care Units (NICUs).

**Objectives:** To scale up and adapt FICare to make it suitable in level IIIC NICUs, which care for extreme prematurity and other complex medical or surgical neonatal conditions.

**Materials and Methods:** Step 1 was the creation of the FICare implementation team (FICare-IT) and baseline analysis of current procedures for critical care to identify needs, wishes, and requirements; we aimed for protocol elaboration tailored to our cultural, architectural, and clinical context (March 2017 to April 2018). Step 2 as a dissemination strategy by FICare-IT acting as primary trainers and mentors to ensure the education of 90% of nursing staff (May 2018 to July 2018). Step 3 involved piloting and evaluation with the aim to refine the procedure (July 2018 to December 2020).

**Results:** A rigorous but flexible protocol was edited. The FICare educational manual included two curricula: for healthcare professionals/staff (Training the trainers) and for families (Education of caregivers), the latter being categorized in two intervention levels (basic and advanced), depending on the infant care needs and parent's decision. In total, 76 families and 91 infants (74.7% preterm; 18.7% complex surgery; 6.6% others) were enrolled in the pilot. No differences in acceptance rate (overall 86.4%) or in the number of infant-family dyads in the program per month were observed when considering the pre- and post-Covid-19 pandemic periods. All families, except for one who dropped out of the program, completed the agreed individualized training. Mothers spent more time in NICU than fathers (*p* < 0.05); uninterrupted time spent by mothers in NICU was longer during the pre-pandemic period (*p* < 0.01). Observed time to reach proficiency by task was within the expected time in 70% of the program contents. The parents revealed educational manuals, workshops, and cot-side teaching sessions as essential for their training, and 100% said they would accept entry into the FICare program again.

**Conclusions:** The principles of the FICare model are suitable for all levels of care in NICUs. Leadership and continuous evaluation/refinement of implementation procedures are essential components to achieve the objectives.

## Introduction

Extremely low gestational age neonates are at risk of developing short and long-term complications that can alter their life course (1–5). This patient group, in addition to other preterm and non-preterm infants who suffer severe neonatal acquired diseases, congenital malformations subsidiary of complex surgery, or rare diseases, sees prolonged hospital stay and faces similar burdens and challenges (6). Together, they can be referred to as high-risk neonates. In this context, parental stress, anxiety, and depression are frequently reported, and these may negatively impact normal bonding and the psychosocial evolution of the individual (1, 7–9). In fact, the power of intense parental stress during the first years of a child's development can be just as important as the biological condition at birth (10, 11). In addition to higher rates of family dysfunctions and economic problems reported, the parents' overprotective reactions toward the vulnerable child, partly motivated by the lack of security in their own abilities and that of the child, hinder the establishment of social relationships and the incorporation of the child into the labor market in adulthood (12, 13). To avoid abiding by these mechanisms, adequate parental training and information are critical (2, 3, 14–18). Therefore, new healthcare models for high-risk newborns are necessary, and these should take into account the global nature of the child, including the care of the family.

Family Integrated Care (FICare) is trademarked by Mount Sinai Hospital in Toronto, and it includes a four-pillar model of care proposed to foster feelings of self-confidence and competence regarding interaction with the child and the ability to be involved in their upbringing (3, 4, 19–23). The FICare model integrates parents into the direct care of their child by having them work together with responsible healthcare personnel who continue to provide medical treatment. To reach proficiency, parents undergo specific training by professionals who act as teachers and guides. Short-term clinical benefits have been reported (23–27) in addition to decreased levels of stress and anxiety in their families (23, 24). The empowerment of parents allows them to feel more secure in caring which in turn will reduce hospital stay and use of emergency services after discharge. Therefore, not only benefits in health are expected but also socioeconomic benefits (28–31). Up to date, reports on FICare model implementation are almost limited to stable preterm infants admitted to NICUs (23–31). However, turning parents into true experts in child care and development, as well as a source of love, protection, and support, is a path that should not be followed without careful planning.

Given the benefits already described, our purpose was to scale up the FICare model by offering two implementation levels (basic and advanced), making it suitable across the whole spectrum of care of the high-risk neonate.

The present report describes the FICare model implementation strategy used in our clinical setting, a level IIIC NICU, and evaluates the methodology followed and the effort used for its development. As one of the most determining elements of the success of the program is the uninterrupted time spent by parents in the NICU, a special focus was placed on analyzing the impact that the Covid-19 pandemic had on the workplan. In addition, at the time the project started, there were not single-family room facilities in our NICU, so that infants remained in open-ward rooms gathering a variable (from 5 to 8) number of patients.

## Materials and Methods

The Department of Neonatology at La Paz University Hospital holds the largest NICU in the Madrid region, and it is one of the largest in Spain. The maternal and child hospital covers a population of approximately 600,000 inhabitants, attends 6,000 deliveries per year, and is a national referral center for fetal surgery, cardiovascular surgery, extracorporeal membrane oxygenation (ECMO), or treatment of retinopathy of prematurity, among other complex processes. Of a total of 67 beds, the NICU has 24 beds for infants on mechanical ventilation plus 15 additional beds for highly dependent non-ventilated infants. More than 200 nurses and nursing assistants (33 per shift) work in the department. There is also active teaching activity among the staff that includes nurse residents (18/year) and students (16/year). The average annual activity at the Department of Neonatology is above 1,500 admissions, and 3,600 visits at the outpatient clinic. The NICU attends all kinds of medical and surgical diseases, has its own Human Milk Bank facilities, and a Royal McDonald House Charities-Family Room.

Our NICU policies allow parents to remain at their infant's cot-side 24 h around the clock. However, the visitors' policies changed during the lockdown period imposed by the COVID-19 pandemic. Accordingly, from mid-March to the end of June 2020 (first wave lockdown), only one visitor per child was allowed to stay for a maximum of 2 consecutive hours at the hospital; from July 2020 to the present, no time restriction was imposed but the number of visitors per infant remained unchanged. Most of the attended population at our NICU is Caucasian or American-Hispanic, with a low proportion of other cultures/ethnicities (mainly Muslims from Morocco).

**Figure 1:**
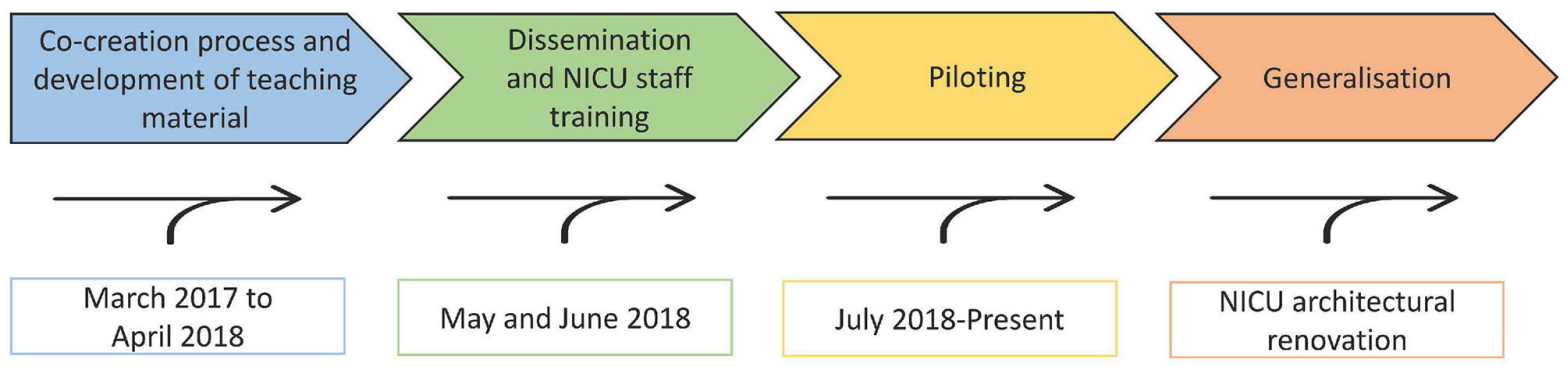
FICare program development and implementation workplan.

The project leader (AP) conceptualized the idea and elaborated a step-wise workplan (Figure 1).

The first step was to create the FICare implementation team (FICare-IT). The FICare-IT gathered members of the local associations of veteran parents and included a variety of professional (social workers, psychologists, speech therapists, and sociologists) and non-professional partners in addition to NICU staff (10% of the nursing staff and four neonatologists). A FICare clinical staff co-ordinator was nominated (MTM). The FICare program development and implementation calendar was agreed upon by the FICare-IT in March 2017 (Figure 1).

FICare-IT conducted an analysis of current procedures for critical care to identify needs, wishes, and requirements. As a result of the analysis, the following components of the FICare program to be implemented were defined: the educational component for staff (nurses and doctors) and family primary caregivers to support their roles; psychological support for parents, with a leading role for veteran parents pertaining to the FICare-IT, or individual interventions by hospital psychologist if requested; and the physical support, which included comfortable cot-side chairs and breast-pumps as well as the facilities provided by the Ronald McDonald Family Room (chill-out area, living-room with TV, kitchen, and complete bathroom with shower and personal locker among others). The procedures and main topics to be covered in the FICare training program, ensuring the diverse needs and challenges were properly covered; the specifications of the tools and materials required for training and for pilot's data gathering; and the questionnaires for experience and psychological data gathering were also decided. Expected deliverables were the FICare implementation protocol (procedures and materials for the overall implementation strategy) and the FICare teaching material.

**Figure 2:**
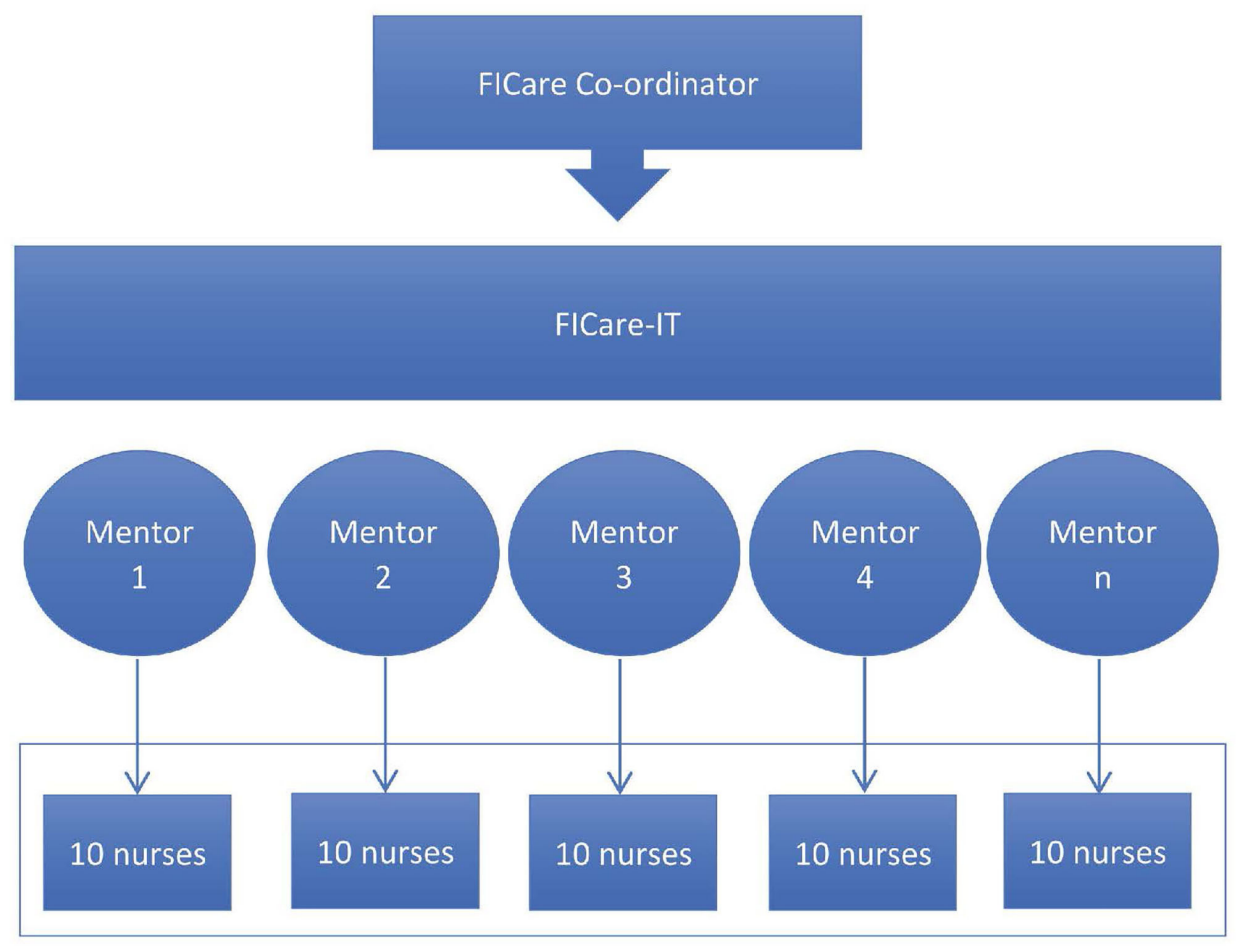
Dissemination strategy at the clinical site following a primary training and mentoring system throughout the whole implementation process.

The second step was the dissemination of the FICare program and training on FICare standards among the NICU clinical staff. The FICare-IT at this stage acted as primary trainers and mentors to ensure that the provision of nurse education covered 90% of active nursing staff in the NICU, as recommended (32). Groups of 10–15 nurses and nurse assistants were created and assigned to a member of the FICare-IT for FICare staff training (Figure 2).

The third step was piloting and evaluation of the FICare program before opening to all potential beneficiaries (a generalization of FICare policies within the NICU); the latter was scheduled for when the new NICU with single-family room facilities opens. Serial FICare-IT meetings were held to evaluate, refine and enrich the preliminary protocol. Barriers and other unforeseen difficulties that were found after a running-in period were taken into account for these purposes.

### Statistical Analysis

The data were analyzed with the statistical program SAS 9.3 (SAS Institute, Cary, NC, USA). Qualitative data are expressed as counts and percentages and quantitative data as mean (SD) or medians (IQR). The pre- and post-Covid-19 pandemic periods during the pilot were defined as July 2018 to March 2020 (21 months) and May 2020 to December 2020 (8 months), respectively. Enrolment during April 2020 was stopped due to lockdown constraints. Non-parametric tests (Mann-Whitney test and Kolmogorov-Smirnov) were used for comparisons. The values were considered statistically significant with *p* < 0.05.

## Results

### Step 1: FICare Implementation Protocol, Training Program and Teaching Material

A rigorous but flexible protocol tailored to the cultural, architectural, and clinical context of our NICU, with two implementation levels (basic and advanced), was edited and approved by the Research Ethics Committee of La Paz University Hospital. The scope of the program covered preterm and term neonates (and their families), admitted to NICU due to complex congenital or acquired diseases or immaturity-related issues, for which a long length of stay was anticipated (i.e., at least three consecutive weeks), providing all inclusion criteria were fulfilled and none of the exclusion criteria applied (Table 1).

**Table 1:** Eligibility and exclusion criteria for FICare program.

**Inclusion criteria for infants** 1. Birth weight at or below 1,500 g or gestational age at or below 34 weeks. 2. Any other peri-neonatal condition anticipating NICU admission for at least 3 weeks. 3. Decision to provide full life support.	**Inclusion criteria for parents** 1. Willingness to spend at least 6 h per day at NICU. 2. Attend educational sessions. 3. Active involvement in care for their infant for at least a 21-day period. 4. No intellectual or language barriers to understanding. 5. Signed informed consent.
**Exclusion criteria for infants** 1. Decision not to provide full life support. 2. Critical illness unlikely to survive. 3. Scheduled for early transfer to another hospital.	**Exclusion criteria for parents** 1. Intellectual handicaps that impede learning-understanding. 2. Language can't be understood by trainer. 3. Refusal to sign the informed consent. 4. Mental, psychiatric problems or under legal supervision.

A strategy for recruitment was established to avoid overloading parents with information during the critical period after birth when they are most vulnerable. Whenever possible, preliminary information about the FICare program was distributed to parents of potential candidates antenatally. Perinatal committees, where complicated pregnancies are discussed among obstetricians and neonatologists, were the ideal forum to comment on potential candidates. An appointment with eligible families was scheduled according to the information gathered in these meetings. In the case antenatal information was not provided, families were approached after NICU admission. Once the families accepted to participate in the FICare program and signed the informed consent, the training tools were provided. A maximum of four infant-family dyads on FICare at a given time was proposed for the pilot. This policy was changed after a 6-month running period of the program when a maximum number accepted was scaled up to 10 infant-family dyads.

The FICare training program was divided into two curricula: for healthcare professionals/staff (Training the trainers) and for families (Education of caregivers), the latter being categorized in two intervention levels (basic and advanced) depending on the infant care needs and parent's decision (Table 2). The following modules were identified as the minimum training contents to be delivered to foster FICare:

- Training the trainers: (1) understanding the boundaries of the FICare model and how to promote FICare among families; (2) psychosocial needs of families (resilience, stress and anxiety, or mourning) and communication skills (assertive communication); (3) how to involve families in NICU (safe conduct in NICU environment, attachment and bonding, how to perform family-centered medical rounds); and (4) professional self-care (burnout and compassion fatigue).
- Education of caregivers: (1) comprehensive description of the FICare model (the strengths and training methodology) and the functional and architectural structure of the NICU; (2) family self-care (stress and anxiety, resilience, or mourning); (3) learning about infants' neurobehavior, stress, and pain; (4) taking part in baby care (basic level), where parents will be “professionalized” to become the first-line care providers of their children; (5) taking part in baby care (advanced level), specific task's training for infants who require even more specialist care; and (6) parents will be prepared for home, and a map of the social resources available at the local setting will be provided.

**Table 2:** “Taking part in baby care”: description of contents by FICare implementation level (basic and advanced), the expected and observed training time, and the number of families certified by task.

**Task**	**Level**	**ETT(days)**	**OTT (median/IQR)**	**n**
**Hand hygiene:** Hand washing rules before and after interacting with their baby or his/her environment.	B+A	2	3 (2–4)	70
**Physiology and monitors:** Parents are trained on the general physiology principles, alarm limits, and running of the medical devices essential for baby care, i.e. incubators, thermometers, scales, or electrodes.	B+A	10	9 (6–12)	63
**Intravenous lines:** To identify the types of lines and the type of infusions used for intravenous administration of nutrition, medication, or blood derivatives. Parents understand the general running of programming flux devices and will be able to report the nursing team any unexpected event.	B+A	3	3 (3–4)	16
**Bathing:** Parents take the responsibility of baby grooming without disrupting their connection with sensors and devices.	B+A	4	3 (2–6)	67
**Breastfeeding:** Mothers are advised and supported on how to improve their own nutritional needs during breastfeeding, breastfeeding techniques and baby positioning, how to foster milk production, manual and pump milk extraction techniques, and mastitis prevention.	B+A	7	6 (4–9)	51
**Other types of feeding:** Parents collaborate in baby nutrition, register feeding times and volumes, double check the milk to be administered, and applying oral sensory stimulation techniques to optimize their baby's sucking and swallowing reflexes.	B+A	5	7 (5–11)	60
**Skin-to-skin & kangaroo method:** Skin-to-skin contact or kangaroo care is provided in almost any condition; parents help the nurse or do themselves the baby's transfer from cot/incubator to mother's/father's chest.	B+A	5	4 (3–8.7)	52
**Dressing & diaper changing:** Can represent a stressful event in the unstable baby. Parents receive guidelines to do these tasks in a safe manner to ensure the baby's comfort.	B+A	2	3 (2–5.2)	66
**Oral medication:** Parents know about the medications prescribed and the indication, the dosage, and the administration route. They only administer oral medications always overseen by the nursing team.	B+A	2	3 (1–4)	41
**Temperature:** Parents daily measure their baby's armpit temperature and report any unexpected value.	B+A	5	3 (2–5)	28
**Mouth and skin care:** Parents follow strict guidelines to ensure mouth and skin care in the tiny body of their babies focusing on monitoring devices connected to their skin by adhesive patches.	B+A	2	3 (2.2–5)	56
**Interaction:** This section focuses on developmental care: measures to avoid stress and promote infant's neurological and emotional development.	B+A	10	6 (4–9.7)	56
**Positioning:** Parents are trained to promote normal muscle development and movement patterns according to the baby's medical conditions.	B+A	14	8.5 (6–11)	56
**Neurobehaviour, stress, and pain:** Parents understand the different neurobehavioral statuses of their baby to guide interactions with him/her in a confident manner. Parents are able to identify signs of stress/pain and learn how to mitigate them (non-pharmacologic analgesia, such as noise and light control, non-nutritive sucking, contention, positioning, etc.).	B+A	14	7 (4.2–10)	56
**Non-invasive respiratory support:** Parents will learn to identify common breathing patterns and apnea events. Also, they are involved in supervising general items in non-invasive respiratory support, notions on supplementary oxygen and adjustments, and placement of ventilator interphase or nasal cannula. Parents understand relevant alarm events.	A	7	7 (4–10)	35
**Naso/orogastric tube:** Parents collaborate with the nursing team in tube positioning. Also, parents double check the milk to be administered and register feeding times as appropriate.	A	3	5 (2–7)	27
**Urinary catheter care:** Parents collaborate with the nursing team in urinary catheter placement and maintenance.	A	14	3 (2–3.7)	12
**Ostomy care:** Parents take care of their baby's stoma with a special focus on skin protection when changing the pouch.	A	5	5 (3–7)	4
**Daily balance:** Parents evaluate and register body and diaper weight, total daily fluid intake, fontanelle and skin status, and vital signs.	A	10	3 (2–4)	47
**Invasive respiratory support:** Parents support their infants while nurses are doing respiratory procedures such as the amount of supplementary oxygen and other basic principles, adjustment of ventilator connectors, or understanding relevant alarm events.	A	14	3 (2.5–5)	9

*ETT, expected training time; OTT, observed training time; B, basic; A, advanced*.

The FICare training tools included an educational manual (14) on all the contents described above provided in both printed and PDF format to caregivers and a notebook to be used by parents for systematic daily data recordings (anthropometry, vital signs, oxygen supply, or nutrition parameters), as well as a space for free text. The training material was ready by April 2018.

### Step 2: Dissemination and Training on FICare Standards Among the NICU Clinical Staff

A teacher education system started in May 2018. The Chief Nurse and FICare co-ordinator defined the mentor-assigned trainee groups and the calendar. Face-to-face meetings took place for 2 months. By June 2018, the program and procedures were disseminated among 90% of the NICU staff (doctors, nurses, and nurse assistants) to become facilitators of FICare. In these meetings, mentors particularly focused on the relevance of training harmonization and the requested adherence to the contents and procedures as described in the educational manual (14). Mentors were available by phone or email to attend to any doubt/request from their respective trainees.

The family training process relayed on three cornerstones that were agreed upon between mentors and the staff facilitators:

- Individualized theoretical and practical learning by tasks: two family caregivers per family were trained through cot-side face-to-face sessions, following an individualized teaching plan adjusted to the baby's clinical condition and the wish of the parents. Once proficiency was fully accredited in a given task, the family caregiver was certified by the training nurse (and registered) and was allowed to do this task autonomously.
- Workshops: family caregivers were invited to attend 45-min open sessions on relevant topics of the learning contents, to express their doubts and concerns, as well as to share their experiences with other families. Three meetings per week were programmed; selected topics were sequentially repeated every 4 weeks.
- Registry of teaching activities and task certifications in the corresponding logbook.

In February 2020 a FICare workshop for all the NICU staff (doctors and nurses/nurse assistants) was organized to refresh the knowledge and share the experiences lived during the previous months of piloting.

### Step 3: Pilot Study and Evaluation of the FICare Implementation Program

From July 2018 to December 2020, 88 families who fulfilled the FICare program entry criteria were approached (57 families pre-COVID-19 pandemic; 31 families post-COVID-19 pandemic); we saw an 86.4% acceptance rate (91.2% pre-COVID-19 pandemic; 77.4% post-COVID-19 pandemic), resulting in a total of 76 parents and 91 infants being enrolled in the pilot. The main neonatal diagnoses of the participating infants were as follows: prematurity (*n* = 68, 74.7%), congenital heart defects (*n* = 13, 14.3%), a variety of genetic syndromes (*n* = 5, 5.5%), congenital diaphragmatic hernia assisted by extracorporeal membrane oxygenation (ECMO) (*n* = 2, 2.2%), kidney dysplasia (*n* = 1, 1.1%), esophageal atresia (*n* = 1, 1.1%), and ileal atresia (*n* = 1, 1.1%). Relevant clinical data of the infants included are displayed in Table 3.

**Table 3:** Clinical features of the infants enrolled in the FICare pilot according to main neonatal diagnosis.

	**Prematurity *N* = 68**	**Other complex conditions *N* = 23**
Postnatal age at enrolment (days), median (IQR)	8 (6–14)	7 (3–15)
Gestational age (wk), median (IQR)	28^3^ (26^1^-30^5^)	38^3^ (37^1^-39^5^)
Birth weight (g), mean (SD)	1114 ± 339	2966 ± 475
SNAPPE-II, median (IQR)	18 (1.25–32)	12.5 (3.75–23)
Invasive MV, *n* (%)	33 (48.5)	22 (95.7)
Days on invasive MV, median (IQR), (min-max)	0 (0–7), (1–81)	10 (5–16), (3–63)
Use of non-invasive MV, *n* (%)	63 (100)	21 (91.3)
Days on non-invasive MV, median (IQR), (min-max)	8 (3–23), (1–76)	11.5 (2.75–22.5), (1–63)
Central catheter, *n* (%)	46 (67.6)	23 (100)
ECMO, *n* (%)	–	2 (8.6)
Parenteral nutrition, *n* (%)	46 (67.6)	23 (100)
Surgery during NICU admission, *n* (%)	10 (14.7)	21 (91.3)

*Invasive MV, intubation and mechanical ventilation; non-invasive MV, high-flow nasal canula or nasal continuous positive airway pressure; SNAPPE-II (33); ECMO, extracorporeal membrane oxygenation*.

The median number of infant-family dyads included in the program per month was 3 (1**–**5) and 4 (2.25**–**5.75) for the pre- and post-COVID-19 pandemic periods, respectively (*p* = 0.237). Systematic surveillance of uninterrupted daily time spent by parents in the NICU started in January 2020. The observed pre-pandemic period revealed that the median time for mothers was 10 (7**–**10.5) h, while the median time for fathers was 7 (5.5**–**10) h. During the post-pandemic period, the median uninterrupted time in the NICU was 7 (4.5**–**8) h and 5 (4**–**8) h for mothers and fathers, respectively. Mothers spent more time in the NICU than fathers irrespective of the period that was observed (*p* < 0.05). Uninterrupted time spent in the NICU differed between periods, being significantly longer for mothers during the pre-pandemic period (*p* < 0.01).

All families enrolled in the pilot completed the individualized training plan that was agreed on enrolment except one, who voluntarily expressed the desire to abandon FICare. Time spent for parents to reach full proficiency to get certification by task is described in Table 2. Family caregivers regularly participated in daily clinical rounds held by the attending physician and the shift nurse, and their comments and suggestions were considered when making decisions.

The median number of parents attending workshops was two (one to three). The main argument given by parents to decline workshop participation was the coincidence with the infants' feeding time.

The results on the satisfaction questionnaire about the FICare program for families are depicted in Table 4. In general, learning material, workshops, and cot-side teaching sessions were judged as essential for their training. Among the experiences described by parents, it is worth mentioning the gain in safety and self-confidence in caring for their children both in the hospital and after discharge:

**Table 4:** The FICare program satisfaction questionnaire for families.

	**Median (IQR)**
Do you think the information about the FICare program has been adequate and complete?	5 (4–5)
Have you been able to answer your questions about the FICare program with ease?	5 (5–5)
Do you think that the teaching book has been useful in your training?	5 (4–5)
Do you think that the notebook has been useful in your training?	4 (3–5)
Do you think that the face-to-face sessions at cot-side were useful in your training?	5 (4.2–5)
Do you think the interactive workshops have been useful in your training?	5 (4–5)
Do you think that FICare training has helped you feel more secure in the care and management of your baby?	5 (5–5)
Do you think that being included in the FICare program has reduced your stress/anxiety about your infant's clinical course?	5 (4–5)
Do you feel more secure with the knowledge got of your baby's environment?	5 (5–5)
Do you think the professional atmosphere around your baby is appropriate?	4 (4–5)
Do you consider noise pollution excessive?	4 (3–5)
Has communication with nurses and nurse assistants been accessible and close?	4 (3–4)
Has communication with your doctors been accessible and close?	5 (4–5)
Is the language used by healthcare professionals adequate for their understanding?	5 (4–5)
Have you been able to participate in the morning medical round?	5 (3–5)
Have you been able to participate in medical decisions about your child?	4 (3–5)
Do you think that FICare training has helped your baby during the hospital stay?	5 (5–5)
Would you accept to participate in the FICare program again?	5 (5–5)
What is your overall assessment of the FICare program?	5 (5–5)

*Rating from 1 to 5, with 1 being the lowest and 5 highest score; response rate: 51 out of 76 families enrolled*.

“*Our perception is that the program helped us to foster discharge; in complex cases, such as the care of our daughter, this training enable parents to feel ready to care (for) the baby and manage all the devices the baby needs, such as oxygen, home monitoring, or feeding by tube. We have also learned how to deal (with) and respond to “scares”; we are now prepared to keep calm and react appropriately when needed. Hav(ing) learned when it is time to ask for help is a great achievement.”*

Another important issue that parents refer to is decreased stress and anxiety as they are really aware pf their baby and his/her context:

“*Knowledge is power. Knowing what our babies are like and how to address their needs was essential to reduce the stress and anxiety we had due to the entrance of our twins; it has even allowed us to enjoy this stage that we have had to go (sic).”*

As a final point of the parents' evaluation, they were asked whether they would accept to enter the FICare program again, to which all the families were aligned:

“*My answer is yes, a thousand times yes. This program was determinant to learn how to deliver the care that my baby needs (sic). The program allowed us to behave as real parents and not mere observers of our baby's life.”*

During the pilot, weekly FICare rounds were held by at least three members of the FICare-IT. During these rounds, direct contact with families currently involved in the training was established. Parents were invited to report any queries, doubts, or complaints about the program. Teaching notebooks and certification registries were collated, and adjustments to the individual teaching plan were carried out. The feedback on the individual progress reported by either parents or professionals involved in the training was used to adjust and refine the workplan to overcome project barriers and strengthen dissemination (Table 5).

**Table 5:** Measures to overcome difficulties to foster FICare during the pilot.

**Barriers to implementation**	**Mitigation strategies**
Nursing staff reluctant to work alongside the model.	Periodic newsletter about the progress; FICare workshop for staff in mid-term of pilot.
Knowledge of parents about procedures generates insecurity in the professionals.	Harmonization and adherence to contents/procedures on educational manual.
Resistance to allowing full autonomy to parents in tasks already certified.	Grouping patients in the same ward.
Transition from NICU to intermediate care represents a halt in parent training.	Grouping patients in the same ward.
Knowledge transfer in the change of nursing shift.	Standardized template to address the individualized teaching plan.
Progress is made in tasks that are not registered/certified. Parents not routinely involved in clinical rounds.	Weekly FICare rounds with responsible nurse/doctor to ascertain individual progress and certification by task and logbook registry.
Rotation of nursing staff that hinders the continuity of the program.	Assignment of trained nursing staff to FICare.

## Discussion

This is the first study reporting on the feasibility of FICare model implementation in a complex, level IIIC NICU, that gathers surgical and non-surgical processes involving both, the preterm and term infant. The scaling up of the FICare model explored in this work included two levels of care: training parents in the basic tasks, as reported previously (23–31), and the advanced level, which introduces additional tasks that pertain to more specialized care, such as respiratory support-related topics, tube feeding, urinary catheter or ostomy care, and daily balance. Therefore, the main strength of this work is to make the principles of the FICare model suitable across the whole spectrum of care of the high-risk neonate. The scope of the impact is very relevant, given that the variety of professionals involved in the care of these patients far exceeds the exclusively neonatal workforce.

Another important achievement of this workplan was developing and implementing the FICare model in an a priori unfavorable architectural configuration of the NICU, as we had no single-family room for NICU admission at the time the project started. Although not a pre-requisite, single-family rooms positively influence the expected FICare health outcomes (25). This kind of facility favors longer uninterrupted time spent by parents at the hospital and promotes interaction with nurses as the private habitat ensures that there are no “observers” in the environment. In spite of these constraints, and although the NICU architectural remodeling was already scheduled, we decided to start piloting the model with the aim of generalizing FICare policies as future routine NICU standard when the new hospitalization area, which will have such facilities, is inaugurated. No doubt that the current availability of a common area for parents, fitted with a kitchen, complete bathroom, chill-out room, and seating area with TV and internet access, has been a key element in supporting the program.

The experience gained during the 30 months of running the project allowed the FICare-IT to make sequential adjustments in procedures to overcome difficulties and barriers that were found. For instance, in the beginning, a maximum of four infant-family dyads on FICare at a given time was proposed: a decision based on avoiding nurse work overload and guaranteeing that the family training and accreditation by task was correct. We also thought that it was easier to group FICare patients in the same ward if the number of families being trained at a time was smaller. As time went by, we realized that this approach was wrong because, even if intended, the reality was that, for different reasons, patients were not “grouped.” In addition, it was realized that many of the teaching activities had more learning inertia if there were more people in the program at any given time. Consequently, the FICare-IT decided to increase the maximum number of infant-family dyads for training by 3-fold.

The COVID-19 lockdown period imposed new visitors' policies at our hospital, limiting the number and the time allowed for visits. In spite of that, enrolment in the pilot was halted only during the peak of the pandemic in our region (April 2020), while training of parents who already were participating in the program continued as scheduled. In fact, no differences in the enrolment rates were observed when the pre- (91.2%) and post-pandemic (77.4%) periods were compared, with a similar number of infant-family dyads in the program per month. Although mothers spent more time in NICU than fathers, irrespective of the period that was observed, our understanding is that the revised visitors' policies during the post-pandemic period justify the larger uninterrupted time spent in NICU by the mothers observed during the pre-pandemic period.

FICare represents a change in people that involve professionals and families as main actors influencing their attitudes, skills, expectations, perceptions, and behaviors. The participation of all health personnel is critical for a successful implementation of a project that promotes a change in the working method and in the established roles. To do so, thorough project planning is essential and should include the development of theoretical and practical support, as well as a good dissemination strategy within the NICU staff. With regards to the first cornerstone, no remarks were raised among the staff regarding the teaching material contents, as expected, because in the educational manual (14) all chapters directly related to how to provide infant care were written by NICU personnel, based on our current protocols, and, as far as possible, adapted to the general population for their better understanding. The feedback obtained from the parents' questionnaires, relative to the educational manual and the notebook, was aligned with that as both got very high scores. In relation to dissemination strategies, continuous action is needed, however. We felt quite “silent resistance” in the different levels of staff, which regrettably has not been systematically evaluated by means of specific questionnaires for professionals. These negative feelings had to be counteracted with a variety of adjustments in the procedures along time, as summarized in Table 5. Increasing the number of participants in the program at a given time, or grouping FICare patients in the same ward for an easier nurse shift programming to optimize the teaching and certification processes, were among the most productive actions taken.

Planning the time needed for task certification is critical, as it is an indicator of the nurse effort required and the moment when parents are allowed to do this task autonomously. We had no a priori experience for this, and our predictions were thus based on the theoretical complexity of the task or how frequently it was to be practiced (Table 2). In 70% of the scheduled tasks, certification was accomplished within the expected time or earlier. In total, 6 out of the 20 blocks of tasks, however, took more time than expected for full accreditation. Most of them relate to common procedures, such as hand hygiene, skin care, or dressing and diaper changes. We suspect that, even though these are “common” routines in NICUs, the fact that our patients were complex and very often clinically unstable showed that more time is needed for parents to overcome fears. It is very interesting, however, that really advanced care, such as naso/orogastric tube care or invasive respiratory-support-related care, took a much shorter time for certification than expected. We think that the explanation for these findings lies in the fact that these tasks always require the participation of the nursing staff, which makes both the professional and the parents feel safer and removes fears.

The way to deliver training to the parents by the nurse staff was acknowledged in general. However, attendance at workshops was much lower than expected. We think this is a weakness of our working plan, as we envisage these open interactive sessions as an opportunity to create good dynamics: a way to heal scars and strengthen knowledge. The justification given by parents was, in general, unsuitable timetables because the sessions were held in a separate room and not at their baby's cot-side. Moving to the admission wards for this purpose prevents gathering a sound number of parents at a given time, and although interaction may be improved (like intimate relations between neighbors), the reaching is lower. The parental feelings about our FICare program, in general, were very positive, and all parents would accept to participate again. Individual comments/remarks were intended to be addressed during the weekly FICare rounds routinely held by members of the FICare-IT. However, it is possible that the items included in the parents' satisfaction questionnaire were leading the participants' responses to some extent.

In summary, this report describes the procedures followed to develop and implement the FICare model at a complex, level IIIC NICU that cares for all kinds of medical and surgical neonatal processes. The adapted protocol scales up the FICare model, introducing two implementation levels: basic (routine tasks addressed in previous FICare experiences) and advanced (specific tasks aimed to prepare parents for infants who require even more specialist care). Our results support that FICare implementation is feasible in this context, even when facing two important adverse conditions: absence of single-family room facilities for hospitalization during the prolonged NICU stay and strict visitors' restrictive policies during the Covid-19 pandemic. Even in the case of thorough planning, continuous follow-up of the protocol procedures and eventual adjustments and adaptations according to the current conditions to solve difficulties raised by both professionals and families are key elements for project success. A limitation of this study is the lack of a systematic assessment of the professional perception of the model. Future studies should address the impact of this adapted scaled-up FICare model on the infants' outcome, the parental stress profiles, the professionals' viewpoint, and the health system economy.

## Data Availability Statement

The raw data supporting the conclusions of this article will be made available by the authors, without undue reservation.

## Ethics Statement

The studies involving human participants were reviewed and approved by Ethics Committee for Human Studies at La Paz University Hospital. Written informed consent to participate in this study was provided by the participants' legal guardian/next of kin.

## Author Contributions

BM-S participated in the development of training material and FICare dissemination-implementation strategy among NICU staff, conceptualized and designed the pilot, and participated in recruitment and data analysis, drafted the initial manuscript, and approved the final manuscript as submitted. MM and MA participated in the development of training material and FICare dissemination-implementation strategy among NICU staff, participated in recruitment and training during the pilot, reviewed the manuscript, and approved the final manuscript as submitted. MS and MC participated in the development of training material and FICare dissemination-implementation strategy among NICU staff, reviewed the manuscript, and approved the final manuscript as submitted. AP conceptualized and designed the workplan and participated in the development of training material and FICare dissemination-implementation strategy among NICU staff, conceptualized and designed the pilot, drafted the initial manuscript, and approved the final manuscript as submitted. All authors contributed to the article and approved the submitted version.

## Conflict of Interest

The authors declare that the research was conducted in the absence of any commercial or financial relationships that could be construed as a potential conflict of interest.
